# Nanoscale copper and silver thin film systems display differences in antiviral and antibacterial properties

**DOI:** 10.1038/s41598-022-11212-w

**Published:** 2022-05-03

**Authors:** Toni Luise Meister, Jill Fortmann, Marina Breisch, Christina Sengstock, Eike Steinmann, Manfred Köller, Stephanie Pfaender, Alfred Ludwig

**Affiliations:** 1 Department for Molecular and Medical Virology, Ruhr University Bochum, Universitätsstr. 150, 44780 Bochum, Germany; 2 Chair for Materials Discovery and Interfaces, Ruhr University Bochum, Universitätsstr. 150, 44780 Bochum, Germany; 3 BG University Hospital Bergmannsheil Bochum, Surgical Research, Ruhr University Bochum, Buerkle de la Camp Platz-1, 44789 Bochum, Germany; 4 Leibniz-Institut für Analytische Wissenschaften - ISAS – e.V., Bunsen-Kirchhoff-Straße 11, 44139 Dortmund, Germany

**Keywords:** Infectious diseases, Materials science

## Abstract

The current Coronavirus Disease 19 (COVID-19) pandemic has exemplified the need for simple and efficient prevention strategies that can be rapidly implemented to mitigate infection risks. Various surfaces have a long history of antimicrobial properties and are well described for the prevention of bacterial infections. However, their effect on many viruses has not been studied in depth. In the context of COVID-19, several surfaces, including copper (Cu) and silver (Ag) coatings have been described as efficient antiviral measures that can easily be implemented to slow viral transmission. In this study, we detected antiviral properties against Severe Acute Respiratory Syndrome Coronavirus-2 (SARS-CoV-2) on surfaces, which were coated with Cu by magnetron sputtering as thin Cu films or as Cu/Ag ultrathin bimetallic nanopatches. However, no effect of Ag on viral titers was observed, in clear contrast to its well-known antibacterial properties. Further enhancement of Ag ion release kinetics based on an electrochemical sacrificial anode mechanism did not increase antiviral activity. These results clearly demonstrate that Cu and Ag thin film systems display significant differences in antiviral and antibacterial properties which need to be considered upon implementation.

## Introduction

Cu and Ag are known as antimicrobial agents for centuries, however, in the medical field these metals have experienced a renaissance over the last years due to the increasing emergence of antibiotic-resistant microorganisms. Beside applications of these metals in numerous consumer products they are used in various biomaterials or healthcare settings to prevent bacterial colonization of implants and devices or to support hospital hygiene procedures to reduce hospital-acquired infections. Especially the pandemic spread of Severe Acute Respiratory Syndrome Coronavirus-2 (SARS-CoV-2) causing the Coronavirus Disease 19 (COVID-19) has exemplified the requirement for effective public health intervention strategies that contribute to controlling virus transmission. However, the development of antiviral surfaces which are able to inactivate adherent virus particles and thereby hinder virus transmission from contaminated surfaces is challenging due to the different inherent properties of microbes compared to viruses.

Both, Cu and Ag, exert broad antimicrobial activities (bacteria, fungi and viruses) and show a low incidence to induce microbial resistance as both attack a broad range of targets in microorganisms^1–3^.The antibacterial activity of Ag is strongly related to the release of Ag ions (Ag^+^) which are formed by oxidative dissolution, while in contrast, zero valent Ag (Ag^0^) exerts no comparable antibacterial activity^2,4–6^. Ag^+^ ions interact with a variety of biomolecules within a cell such as cell membrane and cell wall components, thiol ligands, e.g., sulfhydryl groups of metabolic enzymes, or nucleic acids, and others. Furthermore, reactive oxygen species (ROS) are generated due to Ag^+^ ions which leads to harmful oxidative stress effects^2,7,8^. In general, consequences are biomolecule damage and subsequent cellular dysfunctions which finally inhibit bacterial proliferation up to bactericidal effects. The antibacterial efficiency of Ag can be enhanced by an increase in the Ag^+^ releasing surface area by using e.g., Ag nanoparticles^9^. In addition, recently, we presented a concept to enhance Ag^+^ release kinetics based on an electrochemical sacrificial anode mechanism^10–12^. By combination of Ag with a more noble metal (Au, Pt, Pd, or Ir) within an electrolytic environment (such as biological fluids) the less noble Ag corrodes in favor of the more noble part (it is “sacrificed”). We have demonstrated that such sacrificial anode surfaces exert much higher antibacterial effects compared to pure Ag surfaces with much higher total Ag^+^ due to the electrochemically driven enhanced dissolution of Ag. Even though the antibacterial activity of Ag is well understood very few studies describe the antiviral effects of Ag and most of them deal with Ag nanoparticles^13^. Recently, a virucidal effect of sputtered Ag nanocluster/silica composite on face masks was reported^14^.

In contrast to Ag surface materials the virucidal properties of Cu and Cu alloy surfaces have been studied more frequently^13^. The virucidal effects of Cu rely also on the release of ions and both Cu ionic species (Cu II and Cu I) contribute to the biocidal activity^15–17^.

Remarkably, there is an absence of comparative studies on Cu and Ag opposing their bactericidal and virucidal properties within same experimental setups using equal coating parameters and equal metal ion concentrations to avoid variations in testing conditions. The results of our study are relevant for the design of Cu and Ag containing surface materials with special emphasis on their virucidal activities. Thus, we aimed to analyze the antiviral properties of surfaces coated with Cu or Ag as well as several Ag-based sacrificial anode surfaces including combinations of Cu and Ag for possible synergistic effects and compare antiviral against antimicrobial performance.

## Results

**Figure 1:**
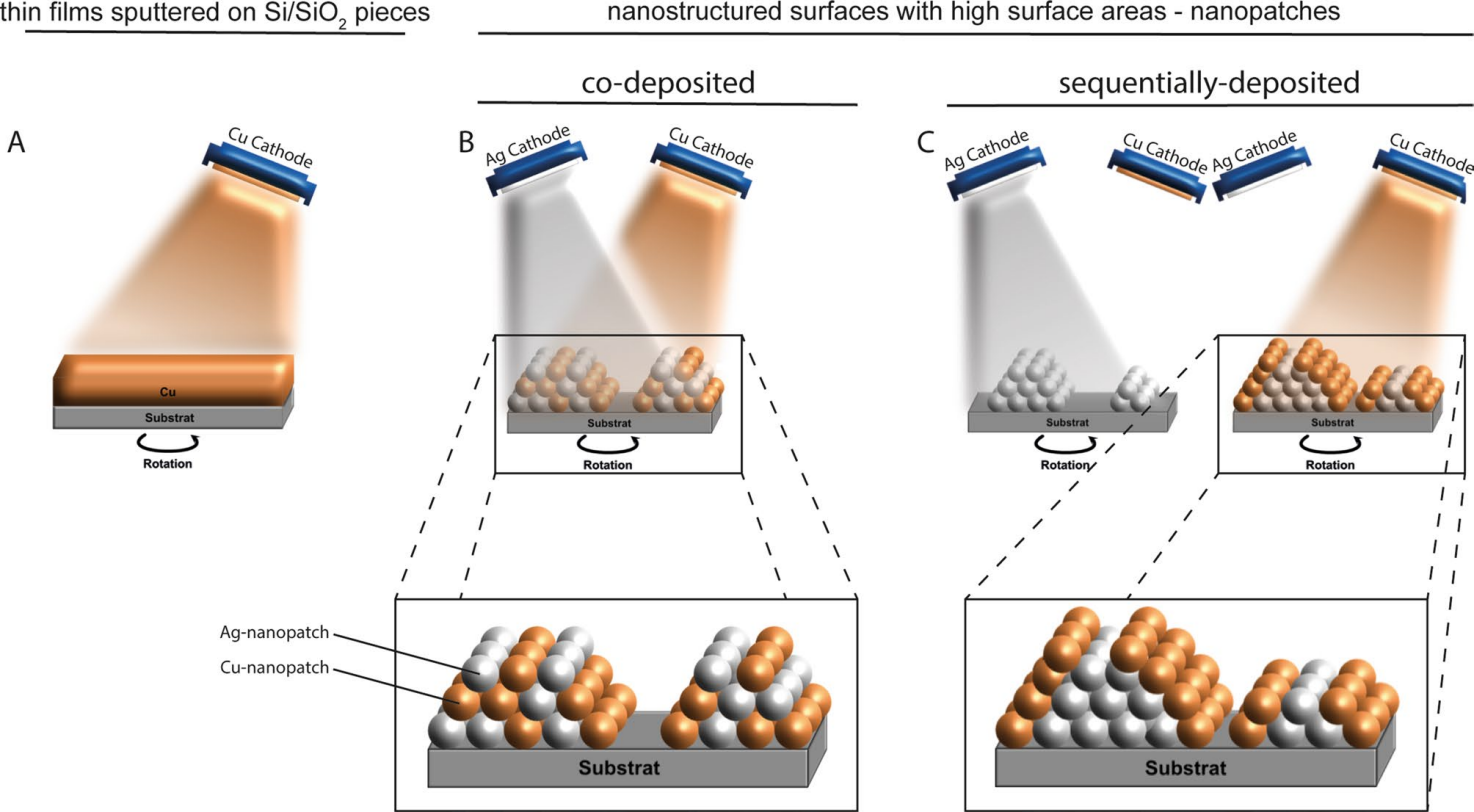
Schematic illustration (not to scale) of the fabrication of Cu and Ag thin film nanostructures by sputter deposition. (**A**) Elemental Cu sputtered homogeneously as a dense and continuous film with 50 nm thickness; (**B**) Cu- and Ag-nanopatches co-sputtered for 120 s; (**C**) Cu- and Ag-nanopatches sputtered sequentially for each 120 s. Nanopatches are island-like nanostructures with nominal thickness < 5 nm.

**Figure 2:**
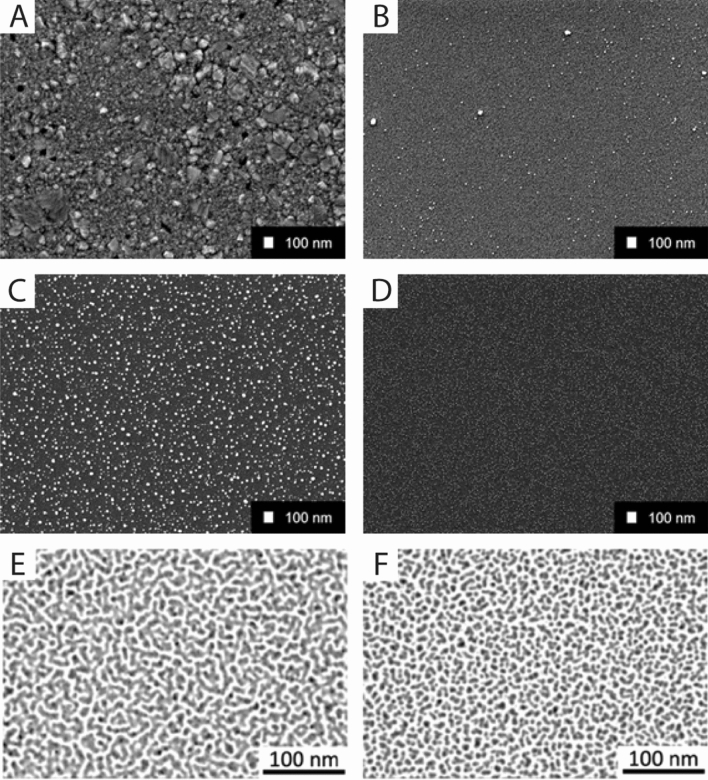
Exemplary electron microscopy images of continuous nanoscale films (**A**, **B**) and nanopatches (**C**–**F**). (**A**) SEM top view of 50 nm Ag, (**B**) SEM top view of 50 nm Cu, (**C**) SEM top view of Ag nanopatches sputtered for 60 s, (**D**) Ag & Pt nanopatches co-sputtered for 60 s, (**E**) TEM image of Ag & Cu nanopatches co-sputtered for 60 s on a TEM grid, (**F**) Cu on Ag nanopatches sequentially sputtered for 60 s on a TEM grid. (**E**, **F**) taken from reference^18^.

The results were achieved using different sputtered thin film surfaces (for details see Experimental Methods): (I) continuous and dense thin films of Ag and Cu (thickness 50 nm) and (II) nanostructured surfaces with high surface areas. The nanostructured surfaces were synthesized by (a) sequentially depositing Ag on Pt, Cu on Ag or (b) by co-sputtering Ag and Pt as well as Cu and Ag. We call the resulting surface structures “nanopatches”, i.e., nanoislands which are formed by the two metals. A schematic workflow is depicted in Fig. 1. In case of sequential sputtering (Ag on Pt, Cu on Ag) the elements tend to be more separated as compared to co-sputtering which tends to mix elements on the atomic scale (Ag & Pt, Cu & Ag) and rather forms an alloy (forced solid solution) compared to the co-existence of elemental films (Ag on Pt, Cu on Ag). The latter is expected to have better sacrificial anode properties. We synthesized nanopatches of two different thicknesses, which offer different volumes of material (e.g., thin Ag/Pt vs. thick Ag/Pt) for releasing metal ions (Table 1). Examples of scanning electron microscopy (SEM) and transmission electron microscopy (TEM) images of the nanoscale films are shown in Fig. 2. The 50 nm thick films are continuous and show different nanocrystalline surface microstructures: the Ag film is rougher and shows larger grains (Fig. 2A) as compared to the Cu film (Fig. 2B). The SEM and TEM images (Fig. 2C–F) of nanopatches (Ag, Ag & Pt, Ag & Cu) show that these nanostructures are discontinuous. They can be regarded as immobilized nanoparticles and thus offer a larger surface area than the continuous films. A detailed TEM investigation of the nanostructure of Ag-Pt nanopatches can be found in reference^12^.

**Table 1:** Thin film surfaces prepared by magnetron sputtering.

Element(s)	Deposition type	Power(s) [W]	Time [s]	Nominal thickness [nm]
Cu		30	630	50
Ag		30	240	50
Cu & Ag	co-deposited	Cu: 12; Ag: 5	60	Cu: 2.4; Ag: 2.4
Cu & Ag	co-deposited	Cu: 12; Ag: 5	120	Cu: 4.8; Ag: 4.8
Ag & Pt	co-deposited	Ag: 5; Pt: 5	60	Ag: 2.4; Pt: 1.02
Ag & Pt	co-deposited	Ag: 5; Pt: 5	120	Ag: 4.8; Pt: 2.04
Cu on Ag	sequential	Cu: 12; Ag: 5	60	Cu: 2.4; Ag: 2.4
Cu on Ag	sequential	Cu: 12; Ag: 5	120	Cu: 4.8; Ag: 4.8
Ag on Pt	sequential	Ag: 5; Pt: 5	60	Ag: 2.4; Pt: 1.02
Ag on Pt	sequential	Ag: 5; Pt: 5	120	Ag: 4.8; Pt: 2.04
Cu		12	60	2.4
Cu		12	120	4.8
Ag		5	60	2.4
Ag		5	120	4.8

In order to qualitatively compare antimicrobial and antiviral properties of sputtered Ag and Cu surfaces, we first evaluated the antibacterial properties of thin Ag/Pt and Ag/Cu sacrificial anode nanopatches. Bacterial tests were performed with *Staphylococcus aureus* (*S. aureus*) using a drop-based experimental setup allowing analysis of planktonic and adherent bacteria. Planktonic bacteria within the drop were quantified by plating on blood agar plates, while adherent bacteria on the sample surface were visualized by fluorescence microscopy.

**Figure 3:**
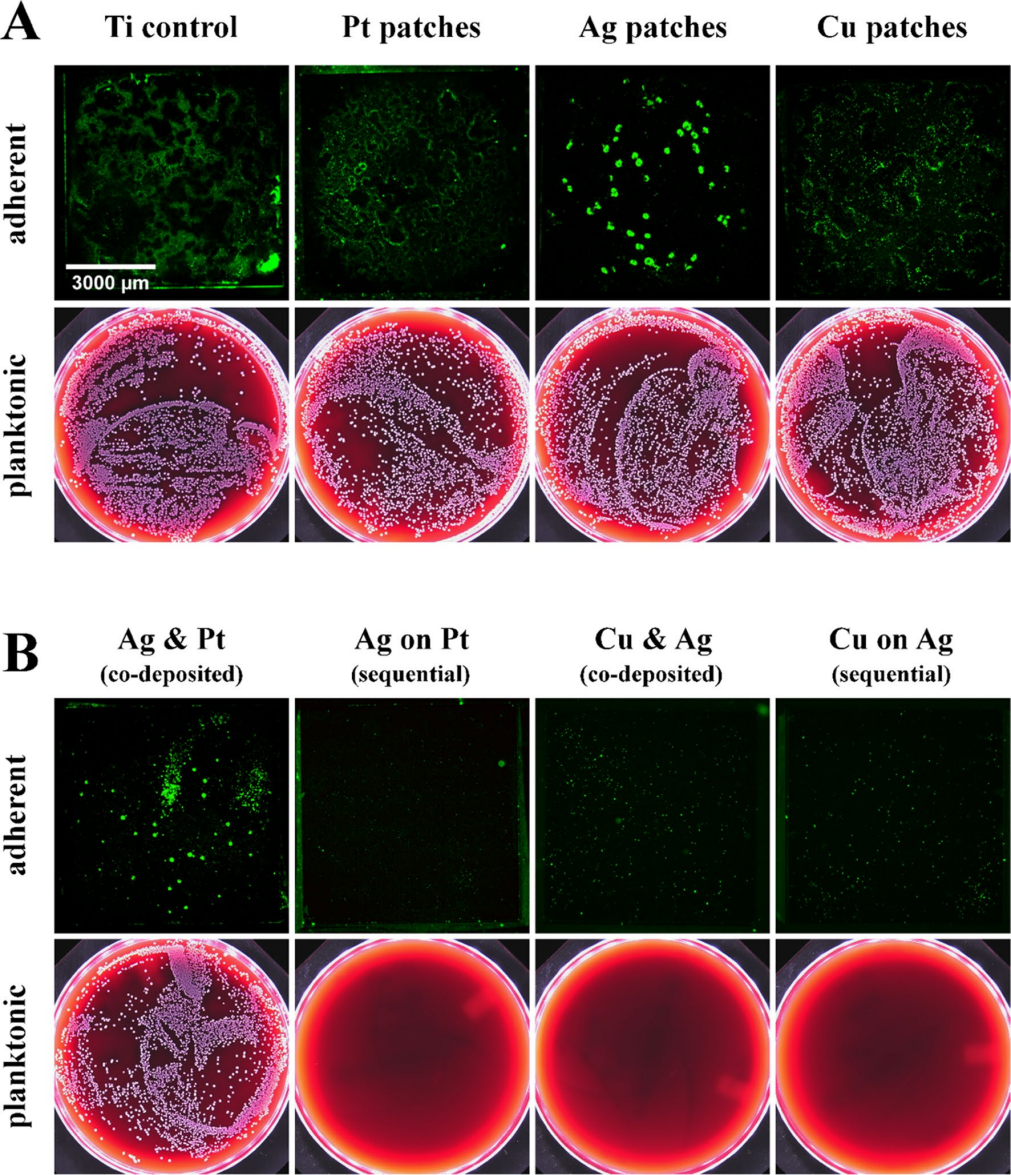
Antibacterial activity towards *S. aureus* (10^4^ CFU/mL) of (**A**) a continuous Ti thin film (Ti control) as well as Pt, Ag, and Cu thin nanopatches sputtered on Ti thin film compared to (**B**) thin Ag/Pt and thin Ag/Cu nanopatches sputtered simultaneously (i.e., co-deposited) or sequentially (first Pt, second Ag or first Ag, second Cu). Sputter time for all samples 60 s. Upper figures: representative fluorescence images of adherent bacteria on sample surfaces after 24 h of incubation and staining with SYTO-9 (green fluorescence); lower images: representative blood agar plates of plated planktonic bacteria in the drop fluid after 24 h of incubation on the different samples (white bacterial colonies indicate viable cells).

Pure Ti thin films as well as thin nanopatches of pure Pt, Ag, and Cu served as controls and exhibited no significant antibacterial activity against *S. aureus* (Fig. 3A). Similarly, bacterial growth was not affected by co-deposited thin Ag/Pt nanopatches indicating the absence of a sacrificial anode effect (Fig. 3B). In contrast, sequentially-deposited thin Ag/Pt nanopatches as well as thin Ag/Cu nanopatches both co- and sequentially-deposited, effectively prevented bacterial growth after 24 h of incubation (Fig. 3B).

**Figure 4:**
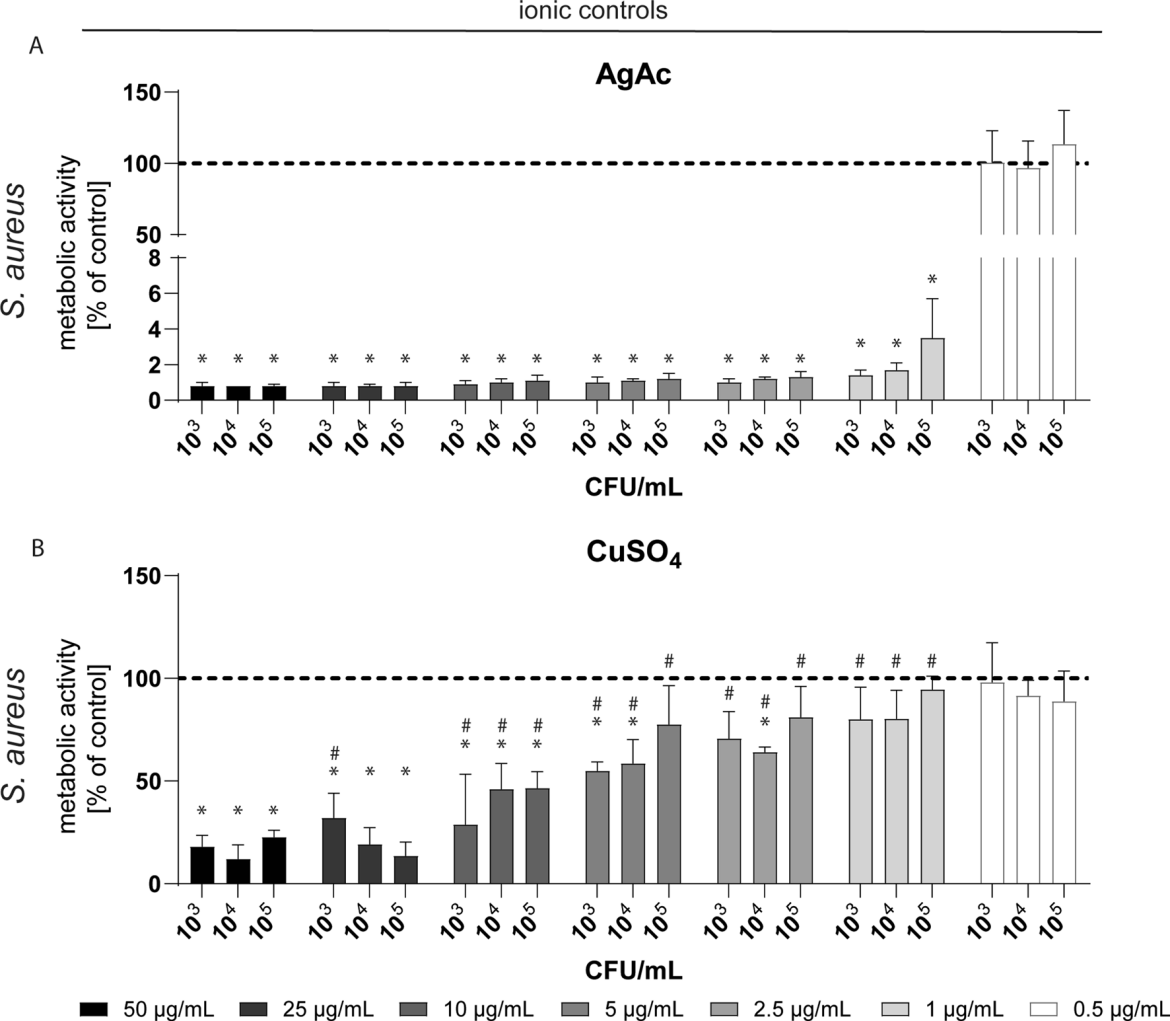
Quantitative analysis of the antibacterial activity of silver acetate (AgAc, panel A) and copper sulfate (CuSO_4_, panel B) solutions towards *S. aureus* (different bacterial concentrations) performed by the AlamarBlue assay. Data are expressed as mean ± SD of at least three independent experiments and given as the percentage of untreated bacteria (no exposure). Asterisks (*) indicate significant differences (*p ≤ 0.05) compared to the untreated control; hash marks indicate significant differences (*p ≤ 0.05) between AgAc and CuSO_4_.

As it is generally accepted that the antibacterial activity of Ag und Cu is strongly related to the release of ions and their interaction with cellular components and processes, solutions of silver acetate (AgAc) and copper sulfate (CuSO_4_) were used as ionic controls for the antibacterial activity of Ag and Cu towards the gram-positive bacteria *S. aureus*^13,14^. Significant antibacterial effects were detected for AgAc at concentrations ≥ 1.0 µg/mL, whereas CuSO_4_ provoked significant effects starting at concentrations ≥ 5.0 µg/mL (Fig. 4).

These results indicate that the absence of antibacterial effects of pure Ag and Cu nanopatches are due to an insufficient ion release from these structures, while the combination of Ag and Pt as well as Ag and Cu leads to enhanced antibacterial activity based on electrochemically driven enhanced dissolution of Ag and Cu, respectively (Fig. 3). Previously, we demonstrated such sacrificial anode effects for nanoparticular and nanostructured Ag/Pt systems^11,12,15^. Regarding the Ag/Cu system, in addition to a possible sacrificial anode effect of Ag on Cu, a combination effect of the two antibacterial metals might be considered^10,12^.

**Figure 5:**
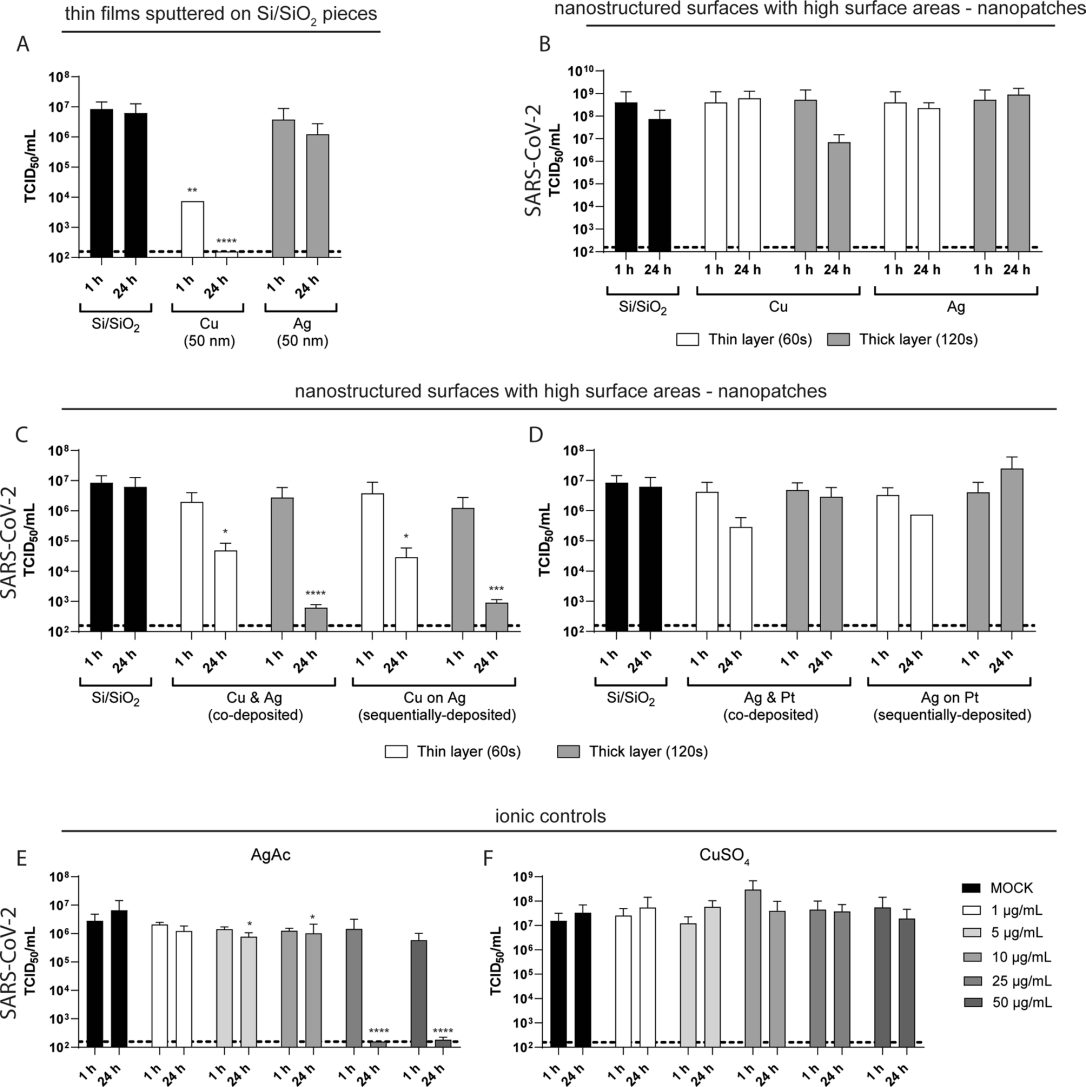
Results of antiviral activity for (**A**) Cu and Ag thin films sputtered on Si/SiO_2_ pieces or (**B**–**D**) nanopatches sputtered on Si/SiO_2_ which were incubated with SARS-CoV-2 for indicated time periods. (**E**–**F**) Silver acetate (AgAc) and copper sulfate (CuSO_4_) solutions, used as ionic controls, were spiked with SARS-CoV-2 an incubated for similar time periods. Residual infectious virus was quantified by TCID_50_ calculation. Dotted line indicates the lower limit of quantification. Data are expressed as mean ± SD of three independent experiments. Asterisks (*) indicate significant differences (*p < 0.05; **p < 0.01; and ***p < 0.001) compared to MOCK (untreated control) or Si/SiO_2_.

After demonstrating antibacterial effects of sputtered Ag and Cu surfaces as well as ionic Ag and Cu solutions, in accordance with previous results, we aimed in a next step to analyze potential antiviral effects of these surfaces against SARS-CoV-2. Viral contamination was mimicked upon inoculation of SARS-CoV-2 onto surfaces for either 1 h or 24 h, before viral titers were determined as TCID_50_/mL (Fig. 5). A pronounced antiviral efficacy was observed for thin Cu films sputtered on Si/SiO_2_ pieces after 1 h and 24 h of incubation reducing viral titers for 3 log_10_ and 4.5 log_10_, respectively, whereas Ag films did not reduce viral titers (Fig. 5A). In contrast, sputtered Ag surfaces only marginally and not significantly affected viral titers upon 24 h exposure. ICP-MS analysis suggested that Cu ions in the range of 10,000–14,200 µmol/L are released from these films during 24 h, indicating that this amount is sufficient for efficient inactivation of SARS-CoV-2 (Supplementary Fig. 1). While Ag nanopatches did not affect viral infectivity, nanopatches with a thick layer of Cu reduced viral titers by 1 log_10_ when incubated together with SARS-CoV-2 for 24 h (Fig. 5B), resulting in an ion release of around 720 µmol/L (Supplementary Fig. 1). Interestingly, Cu & Ag and Cu on Ag nanopatches enhanced the antiviral properties of solely Cu, with a significant antiviral effect after 24 h, despite similar amounts of Cu being released (870 µmol/L, Supplementary Fig. 1). This could be related to the improved electrochemical Cu ion release mechanism in the sacrificial anode structure, where Cu can be easily oxidized and released to the medium as Cu ions, due to the presence of Ag, support the reduction process. Thin layers reduced viral titers by 2.2 log_10_, whereas thick layers increased virus inactivation resulting in reduction factors of 3.9 log_10_ (Fig. 5C). There was no difference in the antiviral effect detected between co- and sequentially deposited nanopatches. This demonstrates that pure Cu films offer the highest antiviral effect (Fig. 5A), which correlates to the highest release of ions (13,000 µmol/L, Supplementary Fig. 1). In contrast, co-deposited and sequentially deposited Ag-Pt nanopatches did not reduce viral infectivity within 1 h incubation. A mild, but not significant antiviral effect (1 log_10_ reduction of viral titers) was observed after 24 h incubation with thin Ag & Pt and thin Ag on Pt nanopatches (Fig. 5D). In order to further examine the observation that Ag-coated surfaces do not affect viral infectivity, we used silver acetate solutions (AgAc) as a reference to test antiviral properties of Ag at higher ion concentrations as compared to what can be released from a thin film surface or a nanopatch structure. We included concentrations ranging from 1 µg/mL up to 50 µg/mL Ag and inoculated the solution with SARS-CoV-2 containing supernatant for 1 h or 24 h before determining viral infectivity as TCID_50_/mL. Only concentrations equal to or higher than 25 µg/mL displayed antiviral properties and completely abolished infectivity of SARS-CoV-2, however, only upon prolonged incubation of 24 h (Fig. 5E). In contrast, virus exposure towards CuSO_4_ solution in a similar experimental setup had no effect on viral titers (Fig. 5F). In conclusion, we demonstrate a clear antiviral effect of Cu-coated surfaces against SARS-CoV-2 within 1 h exposure, whereas Ag-coated surfaces did not influence viral infectivity.

## Discussion

Rapid and effective prevention measures are urgently needed in order to combat microbial and viral diseases. Fomite transmission via contaminated surfaces was described for a variety of microbes, including several viruses and has been discussed in the context of COVID-19. Even though surfaces are believed to play a minor role for the spread of SARS-CoV-2, public health intervention strategies still rely on disinfectant procedures in order to reduce viral transmission. Various surface coatings exhibiting topographically and/or chemically induced antimicrobial activity have been suggested, including nanostructured materials as well as materials containing antimicrobial agents (e.g. antibiotics, antiviral drugs, nanoparticles) such as commercially available antibacterial/antiviral foils, textiles, paints, and many more^19,20^. Although disinfection can be effective in the prevention of infection spread, the biologically active agents of many widely used surface and hand disinfectants might also be hazardous to humans and the environment, especially at prolonged application or misuse. In particular, skin and ocular irritation as well as chemical burns to the respiratory track might occur. Disruption of the normal skin flora, which normally represents a protection barrier to harmful agents, can even enhance the risk of infection^21^.

In line with van Doremalen et al., SARS-CoV-2 was less stable on sputtered Cu surfaces compared to all other thin films of this study^18^. A thicker film led to a more pronounced antiviral effect. Cu nanostructures in contrast reduced the antiviral effect, which can be attributed to the reduced amount of released Cu ions. Interestingly, combining Cu with Ag by either co-sputtering or sequentially depositing Cu on Ag, strengthened the antiviral effects for the nanoscale structures (Cu nanopatch in comparison to Ag–Cu nanopatch) especially after prolonged incubation of 24 h as monitored by limited dilution assays. In contrast, thin films of pure Ag or Ag-based sacrificial anode nanopatches (Ag in combination with Pt) displayed no significant capacity to inactivate SARS-CoV-2 (Fig. 5B–D), despite efficient release of Ag ions. Ionic control experiments demonstrated that a minimum concentration of ≥ 25 µg/mL is necessary to significantly reduce viral titers when incubated for 24 h. Reducing the silver acetate concentration or incubation period completely abolished any antiviral effects (Fig. 5E). This means that only very high concentrations of Ag ions have an effect on SARS-CoV-2. In the past, most of nanoparticles (NPs) used for studies on antiviral activities were made of Ag (for review see^20,21^) For instance, HIV-1 infectivity was inhibited at concentrations of 25 µg Ag/mL and above^19,21^. Recently, Jeremiah et al. reported that Ag NPs (10 nm) were already effective in lower concentrations inhibiting extracellular SARS-CoV-2 at concentrations between 1 and 10 µg/mL^22^, however, it is possible that nanoparticles exert an additional particle effect on viruses after binding such as steric inhibition. Also, a mixture of nanoparticles in solution containing Au-NP (1 µg/mL) and Ag-NP (5 µg/mL) was effective against SARS‑CoV‑2 and influenza viruses, however, this mixture also contained large amounts (60 µg/mL) of ZnO-NPs and additionally ClO_2_^23^. In general, it must be considered that studies with nanoparticles are not directly comparable to studies on thin films (or thin bulk material) due to several facts such as enlarged surface, high ion release, uptake into cells, electrostatic interaction, steric interaction in case of the small particles, and other factors. Our results on thin film surfaces, nanopatches and ionic solutions clearly indicate differences between antiviral and antibacterial activities of Ag. For inactivation of SARS-CoV-2 about tenfold higher concentrations of Ag ions are required compared to efficient antibacterial concentrations. The reason may be related to the difference in the nature of viruses vs. bacteria. As living organisms, bacteria offer much more Ag-sensitive metabolic processes such as energy generation or cell proliferation compared to a virus particle. Furthermore, some bacteria such as *S. aureus* exhibit a significant inherent tolerance of growth at high Cu concentrations^24^. However, a copper sulfate solution up to 50 µg/mL did not inactivate SARS-CoV-2 (Fig. 5F). In our study the ionic Ag and Cu solutions had similar concentration up to 50 µg metal/mL to allow direct comparison. Whereas ionic Ag is antibacterial at concentrations of ≥ 1 µg/mL, ionic Cu is required at much higher concentrations to inhibit bacterial growth (e.g. 10 mM CuSO_4_). Several groups observed similar differences in antibacterial efficiency between Ag and Cu ions^24–26^, however, at lower total ion concentrations due to the water test medium^26^. Thus, at equimolar concentrations Ag ions shows better antibacterial performance compared to Cu ions. Virus inhibition by soluble Cu ions might require higher concentrations above the used maximal values (50 µg/mL) in our experiments.

In contrast to ionic species, antibacterial and antiviral activities of solid Ag and Cu sputtered surfaces have led to different results. Nanopatches of pure Ag neither exhibit antibacterial nor antiviral activities. Obviously, Ag ion release is insufficient under these experimental conditions. To overcome this limited Ag ion release, the more active ion-releasing sacrificial anode surfaces can be used as they exhibit much better antibacterial efficiency even at lower total Ag content^11,12^ as was also observed here for Ag/Pt samples (Fig. 3). However, even these antibacterial Ag/Pt samples failed to induce any antiviral effect which indicates again the need of higher Ag ion concentrations to reach antiviral activity.

Remarkably, our study shows that solid-state Cu either as a dense film or as nanopatches is able to induce antiviral activity, but not solid-state Ag. The antiviral effects are dependent on the total Cu amount (thickness of the sputtered Cu and ion release) and on the time of sample exposure.

It was reported that solid-state cuprous compounds exhibit efficient antiviral activities, whereas those of solid-state Ag are markedly lower^27^. In particular, the inactivation of influenza virus HA and NA surface proteins are affected by the exposure to Ag and Cu^28^. Solid-state Ag is less susceptible than solid-state Cu to surface oxidation under the experimental conditions to release ionic species^29^. It is known that several metabolic products of Cu such as cuprous oxide (Cu_2_O), sulfide (Cu_2_S), or chloride (CuCl) exhibit high antiviral activities and Cu surfaces retain their anti-infective properties even after oxide formation^27,30^.

Although there are numerous reports on antibacterial and antiviral effects of Ag or Cu and their related compounds, a direct comparison of Ag and Cu as performed in our study, including both bacterial and viral data is rarely found and a high variability of the reported study methods makes such direct comparison difficult^31^.

Recently, the rapid inhibition of SARS-CoV-2 on copper-silver (Cu-Ag) nanohybrid surfaces was reported and the authors attributed the role of primary SARS-CoV-2 inhibition to the Cu content^32^. Our results showed that the combination of Cu and Ag (Cu & Ag nanopatches) exerted significantly more antiviral activity than similar nanopatches consisting of Cu or Ag alone. This effect can be attributed to the enhanced Cu ion release of Cu & Ag nanopatches concurrent with a profound decrease in Ag-ion release compared to Ag nanopatches. Apparently, the presence of silver is nevertheless necessary to enable antiviral effects via nanopatches. These findings suggest that electrochemical processes such as a sacrificial anode effect may play important roles. However, the exact mechanisms for this still need to be clarified in further studies.

Taken together, biocidal surfaces could provide constant antiviral and antibacterial efficacy against reoccurring contamination, thus reducing the spread of certain pathogens, given that the surface stays clean and is not used up, whereas surface disinfection has to be reapplied with every contamination^33^. The antimicrobial activity of Cu-based materials and surfaces was demonstrated against different pathogens, including SARS-CoV-2, MRSA (meticillin-resistant *S. aureus*), VRE (vancomycin resistant enterococci), and other nosocomial pathogens, while techniques such as cold-spray coating or Cu-impregnation would circumvent the need to completely replace existing surfaces^34–36^. However, incubation periods greater than 1 h are not applicable for many administrations and prevention measures should therefore be critically evaluated with respect to the targeted pathogen.

## Methods

### Sputter deposition of thin films

Thin film samples were prepared by direct current magnetron sputtering in Ar atmosphere (0.5 Pa) at room temperature on thermally oxidized Si substrates (Si/SiO_2_, 4.4 mm × 4.4 mm), which were placed on a rotating substrate plate. Sputter targets, 2-inch diameter, of Cu (purity 99.99%, EvoChem), Ag (99.99%, EvoChem) and Pt (99.99%, ESG Edelmetall Services) were used. Data on all films are listed in Table 1. The nominal thickness of the films was calculated from the pre-determined sputter rates of the used elements and the indicated power levels. Examples of scanning electron microscopy (SEM) and transmission electron microscopy (TEM) images are shown in Fig. 1.

### Antibacterial tests

Bacterial tests were performed with Staphylococcus aureus (*S. aureus*, DSMZ 1104) obtained from the German Collection of Microorganisms and Cell Cultures (Braunschweig, Germany). *S. aureus* cultures were grown overnight in brain–heart infusion broth (BHI broth, bioMerieux, Lyon, France) at 37 °C using a shaking water bath (JULABO GmbH, Seelbach, Germany) and bacterial concentrations were determined by turbidity measurements (Densichek turbidity photometer, bioMerieux). The adhesion and proliferation of *S. aureus* on the different nanopatch samples were analyzed using a drop-based experimental setup as reported previously^10,11^. Briefly, 30 µL of a bacterial solution in BHI broth containing 10^4^ cells per mL (CFU/mL) were placed in the middle of each test sample followed by incubation for 24 h in a humid chamber (water saturated atmosphere) under cell culture conditions (37 °C, 5% CO_2_). Subsequently, the drops were aspirated, serially diluted (1:10^4^) and plated on Columbia blood agar plates (bioMerieux) for quantitative analysis of planktonic bacteria. Qualitative analysis of the adherent bacteria was performed by SYTO-9 staining (Molecular Probes, Invitrogen, Karlsruhe, Germany) and detected by fluorescence microscopy (BX61 microscope, Olympus, Hamburg, Germany).

Silver acetate (AgC_2_H_3_O_2_, AgAc) and copper sulfate (CuSO_4_) solutions were used as ionic controls for the antimicrobial activity of Ag and Cu, respectively. Each solution was prepared in sterile ultrapure water and normalized to the total metal content (i.e., for example 100 µg/mL AgAc contains 100 µg/mL Ag). Different bacterial concentrations (10^3^, 10^4^, 10^5^ CFU/mL) of *S. aureus* were incubated in BHI for 24 h with different concentrations of AgAc and CuSO_4_ solutions (0.5, 1.0, 2.5, 5.0, 10, 25, 50 µg/mL) in 96-well microplates at a total sample volume of 200 µL under cell culture conditions. Subsequently, quantification of viable cells was performed by the AlamarBlue assay. Therefore, the bacterial suspensions were incubated with 20 µL of the AlamarBlue reagent (Invitrogen) until visible color change and the fluorescence intensity was analyzed at 590 nm by a microplate reader (FLUOstar Optima, BMG LABTECH GmbH, Ortenberg, Germany). The data of the treated cultures (mean ± SD) are given as percentage of the untreated controls (bacteria cultured without AgAc or CuSO_4_).

### Antiviral tests

To evaluate the inactivation capacity of thin films sputtered on Si/SiO_2_ pieces (see Sputter deposition of thin films), 25 µL of SARS-CoV-2 (hCoV-19/Germany/BY-Bochum-1/2020; GISAID accession ID: EPI_ISL_1118929; 8.8 ×10^6^ TCID_50_/mL) was spotted in the middle of each test sample and incubated for 1 h and 24 h at room temperature (22 ± 1 °C) and 32 ± 1% humidity. Virus was recovered by adding 225 µL of Dulbecco’s modified Eagle’s medium (DMEM, supplemented with 10% (v/v) fetal calf serum (FCS), 1% (v/v) non-essential amino acids, 100 IU/mL penicillin, 100 µg/mL streptomycin and 2 mM L-Glutamine). Subsequently, viral titers were determined by an endpoint dilution assay performed on VeroE6 cells (kindly provided by C. Drosten and M. Müller) seeded at 5 × 10^4^ cells/mL in DMEM one day prior titration. The remaining TCID_50_ was calculated according to Spearman and Kärber.

According to the antimicrobial tests silver acetate and copper sulfate solutions were used as ionic controls. Therefore, SARS-CoV-2 was spiked with different concentrations of AgAc and CuSO_4_ solutions (1.0, 2.5, 5.0, 10, 25, 50 µg/mL) and was incubated for 1 h and 24 h at room temperature. Remaining viral titers were again quantified by an endpoint dilution assay followed by TCID_50_ calculation.

### Inductively coupled plasma-mass spectrometry (ICP-MS)

ICP-MS was used to determine the amount of ions released into the medium containing the virus during contact with the surface of interest. Dilutions were adjusted to a calibration range between 1 and 100 ppb, which represents the higher concentration part of the linear calibration curve. In accordance with the calibration measurements, the samples were acidified with 2% HNO_3_. For allowing multiple measurements or to further adjust the dilution of the same sample, 10 mL sample solution were obtained by adding 300 µL of an ultra-pure 69% HNO_3_ (Roth, Supra quality), thorough mixing and filling up to 10 mL to adjust an acid concentration of 2%. The samples were analyzed with an iCAP RQ system from Thermo Fisher Scientific in KED mode.

### Statistics

Statistical analysis of antibacterial effects was performed by one-way ANOVA with Bonferroni post hoc test. Statistical analysis of antiviral effects was performed by two-way ANOVA in a mixed-effects analysis with Dunnet’s multiple comparison. *p *values ≤ 0.05 were considered as statistically significant.

## Supplementary Information

Supplementary Information.

## Data Availability

All data generated or analysed during this study are included in this published article.
