# Supplementary material for: Nanoscale copper and silver thin film systems display differences in antiviral and antibacterial properties
Source: Sci Rep. 2022 May 3;12:7193. doi: 10.1038/s41598-022-11212-w (PMC9063624; doi:10.1038/s41598-022-11212-w)
Supplement: Supplementary file 1 — Supplementary Information. [file 41598_2022_11212_MOESM1_ESM.docx]

**Supplementary Material**


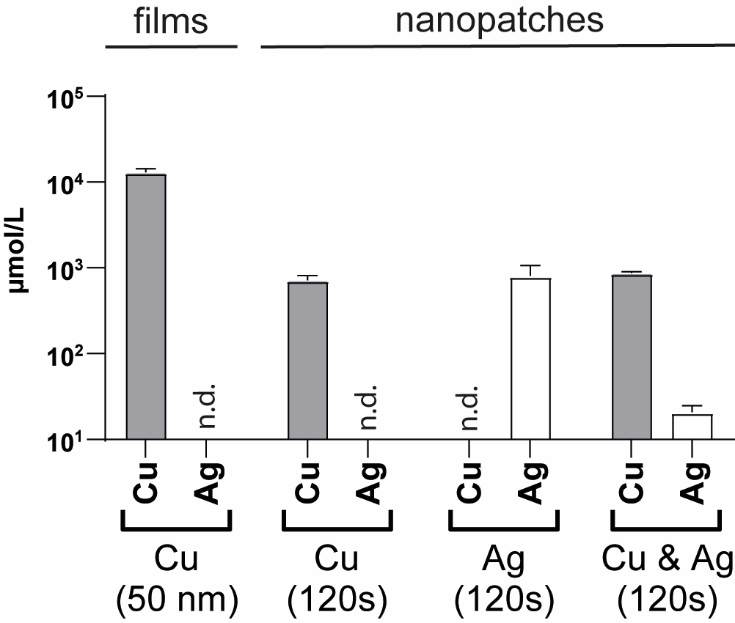


*Supplementary Figure 1: ICP-MS was used to determine the amount of ions released into the medium containing the virus during contact with the surface of interest. n = 5 ± SD. n.d. not detected.*
